# The Medical Student of the Future

**Published:** 1889-11

**Authors:** 


### Topics of the Week.

THE MEDICAL STUDENT OF THE FUTURE.

An editorial in the *British Medical Journal* of Sept. 7th, makes special reference to the student of the future. From it we make the following abstract:

We may assume without the least offense, that most men enter on the study of medicine as the means of obtaining an honorable living; it is not necessary to pretend to any higher motives, like that which should influence a student of divinity. Yet there is room, we maintain, for the influence of the very highest motives in the choice of the healing art as one's calling in life; and the influence is likely to have an actually greater scope, and the end is often even more likely to be achieved, when it is concealed by the friendly covering of the less exalted motive.

There are hundreds of medical men in our land to-day who lead lives of the loftiest enthusiasm, working for the good of their fellow-men, and blessing all within the scope of their influence, pretending with a beautiful humility that they are only following their common business, while they are actually ministering angels. A doctor may live and work for fees and be respected just as any other servant of the commonwealth; he may also live and work for humanity and the love of his neighbor as though he were ordained for the proper work of the ministry. Grand and beautiful as was the work of Father Damien amongst the lepers of Molokai, there is no reason why a medical man should not have done as much, or even more. "Nothing great," says Emerson, "was ever achieved without enthusiasm," and we know it to be so, whatever the hard, cold world may say, which, in truth, does not greatly care for anything which it cannot quite understand.

What is wanted is a noble ideal; given this, it may be exercised as freely in our profession as anywhere in this world of ours. What a man seeks at his medical school this October, that shall he find. Honors, they await him; emoluments, they shall come; happiness, the great enduring pleasure that comes from a sense of duty bravely done, this, too, shall be his, at the price--at a fixed price and no abatement; to this let him make up his mind as quickly as may be. Let him examine himself and know what it is he wants; he can certainly obtain it. Let the lower motive content him; he will not be disappointed. Medicine is rich enough to pay him for his pains; he shall have his house, his servants, and his gig; shall be justice of the peace, mayor of his town, and be held in honor of men. A man, by indomitable energy and perseverance, may get all he wants. As Hazlitt somewhere says, he could always gain admittance to see any famous picture in any great man's home, notwithstanding the darkest frowns of the servants; and he adds that he could, by similar means, have obtained any post under Government which he might have set his whole mind upon.

The thing, therefore, to be sure of at the outset in devoting one's self to medicine, is the end proposed; if self, then not happiness of the highest sort also; if peace of mind and the purest sort of happiness be the end in view, then to live and work for others, for the advancement of the profession in its widest and grandest aspects, is the only certain way to obtain them. Old Thomas Vicary, chief surgeon to St. Bartholomew's Hospital, 1548-62, says in his curious *Anatomie of the Bodie of Man*, that the doctor must be learned, must know his principles, be seen in natural philosophy, in grammar, must speak congruity in logic, speak seemly and eloquently, know things natural and non-natural, and, above all, be good-looking, for whose face is not seemly, it is impossible for him to have good manners.

All this implies much more than is demanded for the mere cramming up for professional examinations. Be liberal in your treatment of the most liberal of all professions, and give at least as much as you take. How few men ever think of paying the least fraction of their indetbedness to science! They consider this all arranged for in their hospital ticket. Such men

> Know, not for knowing's sake;
> Know, for the gain it gets, the praise it brings,
> The wonder it inspires, the love it breeds.

No man striving only for his own happiness can ever attain it, because he is in the midst of forces contending against him, set in motion by every other man of the same determination.

Count Tolstoi has admirably explained why this struggle for individual happiness must necessarily fail of its purpose; and Mr. Browning, in his magnificent poem of "Paracelsus"--which should be known by heart by every medical student of the thoughtful sort, has pointed out how mere knowledge for a selfish end can never bring happiness, whatever else it may achieve; but that love, allied to knowledge, can transform the soul to God-like beauty. We may long, like Paracelsus in the poem,

To wring from Heaven some wondrous good for man; but it may not be given to many of us to do great things. Of every medical man, however, Society—having in view his great endowments, his privileges, his public estimation, and the dignity of his calling—has the right to expect maintenance of the fabric, if not its adornment; and, as he is necessarily looked up to as a "doctor," that he shall be a real teacher how to live the highest mental as well as the healthiest bodily life. As Sir J. Crichton Browne pointed out recently, this can be best done by imbuing our own minds with the prolific and ennobling thoughts of the wisest writers of the past and present; for to teach we must learn. Some will say we ask too much of the overtaxed medical student. Not so. The mental enlargement we demand can be had as relaxation between the intervals of necessary studies. One hour a day with the great poets and prose writers will enable the student to do better work in the medical school; he will not lose his time by this form of dissipation; and when he goes into the great world which lies outside his hospital, he may find his own medicine and that of his patients in the balm for troubled spirits which the philosophers and poets of all time stand by to minister. We say he will not lose his time by this expansion of his education; it will serve to digest his technical knowledge, to combine and blend his studies into a truer and sounder learning than can be tested by examination papers or rewarded by degrees.

Let no student, therefore, think that so much anatomy, physiology, medicine and surgery, signed up and certified for at school and college, suffices to make the medical man of to-day. In one of the most suggestive of the inaugural addresses delivered in London last October, that by Dr. William Ewart at St. George's Hospital, this point was strongly emphasized. We can not refrain from recalling a sentence or two. "Among the youths," says Dr. Ewart, "who elect to follow this calling, many do so in ignorance of what the choice implies. Of no other profession is it more true that an easy entrance examination is unkind. Ours, nowadays more than ever, is an exacting profession. Although neither genius, nor brilliancy, nor even talent are wanted, she claims energy, physical and mental, capacity for sustained efforts, earnestness, and a high moral tone."

A medical practitioner whose whole life is not that of a persevering student has no business in the profession. A man who knows nothing but what his curriculum enforces, and who makes haste to forget that as soon as it has obtained him his license to practice, can only bring discredit on the high-minded and cultivated men who spent their lives in making smooth the path he has unworthily trodden. If medicine is to hold its high position and retain the respect in which it is justly held, the men who are coming forward for its emoluments and distinctions must be equipped with all the richer learning which is required to enable them to hold their own in a world which is daily becoming more highly cultured, and which will certainly demand more from its medical advisers. A mental outlook bounded by six-ounce bottles and an intellect from which there is gradually fading the scanty lore gathered at the medical school with much pain and but partial comprehension far too generally characterize the middle-aged general practitioner of to-day. He of to-morrow will need to know more, and to know it in quite another way.

---

A Case of Senile Dentition.—Dr. T. M. Greenwood, of Mineral Bluff, Ga., reports a very interesting case of senile detention. Patient, female; aged seventy-eight years; mother of twenty-one children at full term. Had never taken a dose of medicine from a physician until June of this year, when Dr. Greenwood was called in and found her suffering with an attack of summer diarrhœa, which lasted for about two weeks. Three weeks thereafter she cut two middle upper incisors. The gums are much swollen, and there is every indication that she will cut a full set of teeth.—*Atlanta Med. and Surgical Journol.*

